# Congenital Hypofibrinogenemia: Presentation of a Rare Coagulation Disorder

**DOI:** 10.7759/cureus.12397

**Published:** 2020-12-31

**Authors:** Karol Acevedo Viales, Kathia Valverde Muñoz, Daniela Gutiérrez Valverde

**Affiliations:** 1 Hematology, Hospital Nacional de Niños, San José, CRI; 2 Medicine, Universidad de Ciencias Medicas, San José, CRI

**Keywords:** hypofibrinogenemia, bleeding, fibrinogen

## Abstract

Fibrinogen is a precursor of fibrin, which acts as a procoagulant plasma soluble protein. It is involved in blood viscosity and clot stability to help in the recovery of damaged blood vessels. We describe the case of a preterm newborn who presented with abdominal distension and manifestations of bleeding in venipuncture sites. In the initial laboratories, prolonged coagulation times were observed with a decreased concentration of fibrinogen. This newborn had transfusion support, with recovery in factor levels and a subsequent decrease in them. Based on this trend and ruling out other possible causes of hypofibrinogenemia, such as liver disease, sepsis, or disseminated intravascular coagulation, the diagnosis of congenital hypofibrinogenemia was made. This case report includes the diagnostic and therapeutic approach of an unusual hemorrhagic presentation in the newborn, highlighting the need for transfusion and dynamic fibrinogen replacement to prevent complications and seek rapid improvement in symptoms.

## Introduction

Congenital fibrinogen (factor I) deficiency is a rare hereditary coagulation defect, with its prevalence difficult to establish because of the large number of asymptomatic cases. It is estimated that congenital fibrinogen disorders correspond to 8% of all rare bleeding disorders. Fibrinogen is a plasma glycoprotein synthesized in the liver and is involved in the final step of the coagulation cascade. The diagnosis is determined by demonstrating decreased activity and/or low levels of immunoreactive fibrinogen in plasma [1].

Disorders of fibrinogen can be inherited or acquired. Congenital forms are typically autosomal recessive. In the case of afibrinogenemia, the affected individuals are homozygous or compound heterozygous for mutations in the gene encoding the fibrinogen, whereas congenital hypofibrinogenemia is often seen in heterozygous carriers for these mutations. The symptoms vary depending on the level of fibrinogen in plasma [1,2]. Associated manifestations include gastrointestinal bleeding, umbilical cord bleeding, ecchymosis, hematomas, hemarthrosis, and central nervous system (CNS) bleeding, among others.

In order to describe such an infrequent pathology, we present a clinical case of hypofibrinogenemia, as well as its clinical course and management.

## Case presentation

**Figure 1:**
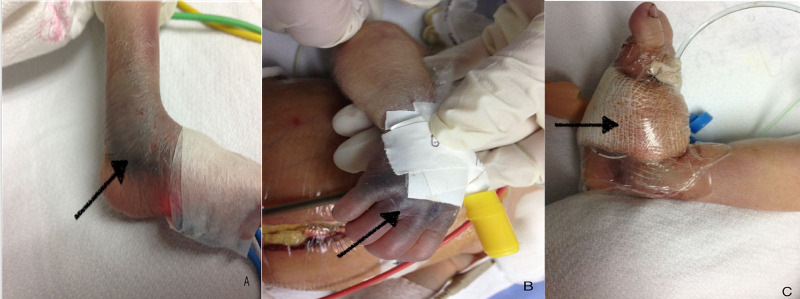
The patient presented with bleeding mainly in venipuncture sites.

A preterm newborn with three hours of life was admitted to the Neonatology Service to be evaluated for abdominal distention. The newborn of a 27-year-old mother with 34 weeks of gestational age, due to the threat of pre-term delivery, had induced lung maturation four days prior to delivery. The baby went through vaginal delivery, with an Apgar score of 7-7, birth weight of 1,970 g, height of 41 cm, and head circumference of 29 cm. He was classified as a preterm newborn suitable for gestational age. At the time of admission, the mother omitted any significant inherited family history. During initial evaluation, mild respiratory distress was documented due to abdominal distension, inaudible peristalsis, and a tense abdomen with an ascitic wave. Ecchymosis was also found at venipuncture sites, with bleeding that did not cease despite mechanical pressure (Figure 1).

An abdominal ultrasound was performed with findings suggestive of intestinal atresia as the first possible diagnosis. The patient’s laboratory workup was as follows: hemoglobin of 10.2 g/dL, hematocrit of 28.6%, mean corpuscular volume of 92.3 fL, mean corpuscular hemoglobin of 32.9 pg, leukocytes of 21,250 x 10^3^/uL, platelets of 110,000 x 10^3^/uL, prothrombin time (PT) of 43% (23.7 seconds), activated partial thromboplastin time (aPTT) of 47.2 seconds, and D-dimer 0.1 ug/mL. The first fibrinogen level (at eight hours of life) was 60 mg/dL. At this time, the patient presented profuse bleeding from all venipuncture sites; hence, it was decided to transfuse first with fresh frozen plasma (FFP). Since the bleeding did not stop, cryoprecipitates were given, which finally controlled the bleeding and made it possible for the patient to undergo the surgical intervention without complications.

At this point, the hematology service was consulted, and discussion with the mother revealed that she had two children in replacement treatment with cryoprecipitates and were followed up in the hematology clinic. With this information, hereditary congenital hypofibrinogenemia was established as a diagnosis.

## Discussion

Clotting factors do not cross the placental barrier, and the fetus initiates the hepatic production of procoagulant and anticoagulant factors at approximately five weeks of gestation; therefore, all clotting factors are present at birth. At 20 weeks, clotting factors are detectable in fetal plasma in low amounts. Therefore, premature infants have even lower levels of factors compared to term infants, but these increase rapidly, with most components reaching adult levels by six months of age. At birth, the only procoagulant factors that are within the adult range are fibrinogen, factor V, and factor VIII [3]. In our case, the decrease in factor I levels in the first hours of life should orient us toward a congenital deficiency. In this case, a prolongation of PT and aPTT was observed, which might be seen in a fibrinogen deficiency, and was confirmed by obtaining a decrease in antigenic fibrinogen as a confirmatory method.

It is important to rule out secondary causes of deficiency in this factor, such as liver disease, hypoalbuminemia, and consumptive coagulopathy, to consequently verify the diagnosis of congenital hypofibrinogenemia, as well as other factor deficiencies.

Fibrinogen plays a key role in the clotting process. It functions not only as the precursor of the fibrin network that gives structure to the clot but also as a promoter of platelet aggregation, fibrinolysis, angiogenesis, is an acute-phase reactant, and participates in other biologic processes [1]. The genes encoding fibrinogen chains are located on chromosome 4; this complex glycoprotein comprises three pairs of polypeptide chains, designated as $\alpha$, β, and 𝛄, and is the main ligand for platelet integrin IIb/IIIa during aggregation. Partial proteolysis of fibrinogen by thrombin allows polymerization to form the fibrin clot [2].

Within the clinical setting of hypofibrinogenemia, it is divided into three different categories: (A) mild, with levels between 1 and 1.5 g/L, which are usually asymptomatic since it is high enough to provide protection against bleeding, (B) moderate, with levels between 0.5 and 0.9 g/L, and (C) severe, with levels less than 0.5 g/L. The last two present a heterogeneous clinical course, with manifestations usually due to hemostatic challenges or even spontaneous bleeding in severe cases [2]. Hypofibrinogenic and dysfibrinogenic patients are often asymptomatic and therefore underdiagnosed.

This difference in presentation could be attributed to the von Willebrand factor, which binds to the glycoprotein complex in platelets and provides a backup mechanism for platelet aggregation, particularly by replacing fibrinogen [4].

According to the International Society on Thrombosis and Haemostasis (ISTH) 2018 classification for congenital fibrinogen disorders, not only the level of factor but also the clinical phenotype is considered. It is important to use a bleeding score to determine the tendency towards bleeding, guide management, and prevent associated complications [2].

Replacement therapy is the current basis of treatment. Different products are available, FFP, cryoprecipitates, and fibrinogen concentrate, with the latter being the treatment of choice [5]. The use of blood components involves a possible association with transfusion reaction, volume overload, and transmission of viral agents; although the risk is low, these effects can be minimized by using factor concentrate [6-9].

The goal when managing the pediatric patient is the prevention and cessation of bleeding. Not all patients require treatment since most are asymptomatic.

In our country, factor concentrates are not available, and therefore using cryoprecipitates as an alternative implies a higher concentration of fibrinogen in a smaller volume compared to FFP. Cryoprecipitates contain 200-400 mg of fibrinogen, which is a similar amount to that found in human plasma, in volumes of 10 to 20 mL [5]. The half-life of fibrinogen is approximately three to five days, with a minimum hemostatic level of 50-100 mg/dL; for this reason, the dose must be administered to achieve this range.

In recent years, advances have been made to define molecular mechanisms and the epidemiology of the disease in cases in which there is a suspicion of a hereditary disorder as well as a hereditary history. Using the sequencing of fibrinogen genes is an appropriate tool; however, these tests are not available in all laboratories and therefore are not required for diagnosis. This leads to the importance that once the patient is diagnosed, genetic counseling is provided to the parents in order to make informative decisions about the disease.

## Conclusions

In conclusion, this case report includes the diagnostic and therapeutic approach of an unusual hemorrhagic presentation in the newborn. It seeks to provide information on such a rare pathology to improve the diagnosis and management of this disease, highlighting the need for surgical treatment in this patient, and therefore transfusion and dynamic fibrinogen replacement to prevent complications.
